# Safety and Immunogenicity of a Modified Self-Amplifying Ribonucleic Acid (saRNA) Vaccine Encoding SARS-CoV-2 Spike Glycoprotein in SARS-CoV-2 Seronegative and Seropositive Ugandan Individuals

**DOI:** 10.3390/vaccines13060553

**Published:** 2025-05-23

**Authors:** Jonathan Kitonsa, Jennifer Serwanga, Hannah M. Cheeseman, Andrew Abaasa, Jane Frances Lunkuse, Eugene Ruzagira, Laban Kato, Florence Nambaziira, Gerald Kevin Oluka, Ben Gombe, Sembera Jackson, Joseph Katende Ssebwana, Leon R. McFarlane, Sarah Joseph, Benjamin F. Pierce, Robin J. Shattock, Pontiano Kaleebu

**Affiliations:** 1 Medical Research Council, Uganda Virus Research Institute & London School of Hygiene and Tropical Medicine Uganda Research Unit, Entebbe P.O. Box 49, Uganda; jennifer.serwanga@mrcuganda.org (J.S.);; 2 Uganda Virus Research Institute, Entebbe P.O. Box 49, Uganda; 3 Department of Infectious Disease, Imperial College London, Sir Alexander Fleming Building, South Kensington, London SW7 2AZ, UKleon.mcfarlane@resolution-tx.com (L.R.M.); s.joseph@imperial.ac.uk (S.J.); benjamin.pierce@imperialcollegehealthpartners.com (B.F.P.);; 4 Department of Infectious Disease Epidemiology, London School of Hygiene & Tropical Medicine, London WC1E 7HT, UK

**Keywords:** self-amplifying RNA (saRNA) vaccine, SARS-CoV-2 immunogenicity, COVID-19 vaccination in Africa, neutralising antibody responses, spike-specific IgG antibodies, vaccine safety and reactogenicity, seronegative vs. seropositive immune responses, phase 1 clinical trial

## Abstract

Background: The COVID-19 pandemic highlighted the need for innovative vaccine platforms that elicit durable immunity. Self-amplifying RNA (saRNA) vaccines offer rapid production and dose-sparing advantages over traditional mRNA platforms. In Uganda’s first SARS-CoV-2 vaccine trial (NCT04934111), we assessed the safety and immunogenicity of a saRNA vaccine encoding the SARS-CoV-2 spike (S) glycoprotein in seronegative and seropositive adults. Methods: This non-randomised phase 1 trial (December 2021–April 2022) enrolled 42 healthy adults (18–45 years), including 12 seronegative and 30 seropositive for SARS-CoV-2. Participants received two 5 μg doses of saRNA vaccine, four weeks apart. Reactogenicity was assessed using diary cards for seven days post-vaccination, and adverse events were monitored throughout the 24-week study. Binding and neutralising antibody levels were quantified using ELISA and pseudovirus neutralisation assays. Findings: The vaccine was well tolerated, with only mild-to-moderate adverse events, including fatigue, headache, and chills. No serious vaccine-related events occurred. Among seronegative participants, 91.6% seroconverted after two doses (median S-IgG: 3695 ng/mL, *p* < 0.001). In the seropositive participants, S-IgG rose modestly from 7496 to 11,028 ng/mL after the second dose. Neutralising titres increased modestly across WT, BA.2, and A.23.1 variants, with no significant differences between groups. Conclusion: The saRNA SARS-CoV-2 vaccine was safe and immunogenic, inducing robust spike glycoprotein-specific antibody responses, particularly in seronegative participants. This trial demonstrates the potential of saRNA vaccines for broader use.

## 1. Introduction

The COVID-19 pandemic profoundly affected livelihoods, health, and economies globally [1,2]. The World Health Organisation (WHO) declared it a public health emergency of international concern (PHEIC) on 30 January 2020, a status that remained until 5 May 2023 [3]. Although the PHEIC status was lifted, COVID-19 remains a global health threat [3]. As of 1 December 2024, approximately 777 million cases and 7 million deaths had been reported [4]. Despite occasional surges, COVID-19 incidence and mortality have declined. While early public health interventions such as lockdowns contributed to containment efforts, vaccination has played a pivotal role in reducing infection with SARS-CoV-2 infections and COVID-19-related mortality [5,6]. To date, approximately 13.6 billion vaccine doses have been administered globally [4].

The Pfizer–BioNTech COVID-19 vaccine received emergency use authorisation in December 2020 and the United States Food and Drug Administration (FDA) approval in August 2021 [7], becoming the first authorised RNA vaccine. This milestone paved the way for rapid approval of subsequent vaccines [8,9]. Accelerated development and deployment were driven by advanced technology, existing infrastructure, and prior research on related viruses, such as the Middle East respiratory syndrome coronavirus (MERS-CoV) [10]. Although vaccination has significantly reduced COVID-19 incidence and mortality, SARS-CoV-2’s rapid evolution diminishes the effectiveness of existing vaccines. Moreover, immune responses from existing vaccines wane over time [11,12,13,14]. This highlights the continued need for COVID-19 vaccine research [15,16].

Since the onset of the pandemic, Africa has faced challenges such as limited research participation, slow vaccine rollout, and low uptake [9,17]. The pandemic’s impact was exacerbated by a weak healthcare infrastructure [18]. Despite a pressing need for vaccines, African countries had minimal involvement in COVID-19 vaccine research and development. Evaluating vaccines across diverse demographics is crucial, as immune responses vary by factors such as race [19], geography [20,21], and local immune microenvironments shaped by chronic infections and inflammation [21].

The self-amplifying RNA (saRNA) vaccine developed at Imperial College London was among the earliest SARS-CoV-2 vaccines evaluated in Africa. Its ability to self-amplify within cells allows for smaller doses, potentially facilitating expanded coverage and reducing production costs [22,23]. This vaccine demonstrated excellent safety and immunogenicity in non-human primates [24] and in phase 1/2a “COVAC1” trials in the United Kingdom [25,26]. In Uganda, the COVAC Uganda trial evaluated a second-generation saRNA vaccine encoding the SARS-CoV-2 spike glycoprotein in SARS-CoV-2 seronegative and SARS-CoV-2 seropositive participants at the MRC/UVRI & LSHTM Uganda Research Unit in Masaka, Uganda. This version featured a vector modification incorporating an ORF4 motif to reduce innate immune responses to the vector (Supplementary Information S1).

## 2. Materials and Methods

### 2.1. Study Design, Setting, and Population

This single-centre, non-randomised phase 1 clinical trial assessed the safety and immunogenicity of a lipid nanoparticle–new corona virus saRNA (LNP-nCOV saRNA-02) vaccine, administered intramuscularly (IM) at 0 and 4 weeks. Eligibility criteria included age 18–45 years, willingness to provide informed consent, and adherence to contraception requirements: female participants agreed to using highly effective contraception, while male participants committed to avoiding pregnancy with their partner from screening until 18 weeks after the second injection. Participants were required to avoid all vaccines, including COVID-19 vaccines, from four weeks before the first dose until four weeks after the second. Those seeking Ministry of Health-recommended vaccines thereafter received appropriate information and referrals. Participants were also required to adhere to the 24-week visit schedule, document reactogenicity events in vaccine diaries, provide required samples, and grant access to trial-related and medical records. Details on eligibility criteria, screening, and enrolment are available in a previously published paper [27].

### 2.2. Procedures During the Screening Period

Screening was conducted within 42 days before enrolment. The schedule of study procedures is summarised in Supplementary Information File S2. Participants received written information about the product, trial design, and data collection in English or Luganda and had the opportunity to ask questions. Those who agreed to participate provided written consent, completed a screening questionnaire, and provided samples for screening investigations.

Data were collected on demographics, medical history, and current medications. Information on contraception use was collected to assess pregnancy risk. Screening included measurements of vital signs, weight (kg), height (cm), oxygen saturation, lymph node evaluation, and skin inspection for severe eczema. A comprehensive respiratory, cardiovascular, abdominal, and neurological examination was performed.

Blood samples were collected and analysed for complete blood count (haemoglobin, lymphocytes, neutrophils, platelets) and biochemistry [creatinine, aspartate transaminase (AST), gamma-glutamyl transferase (GGT), alanine transaminase (ALT), alkaline phosphatase (ALP), total bilirubin, and non-fasting glucose)]. Additional tests included tests for SARS-CoV-2 antibodies, SARS-CoV-2 antigen (if COVID-19 was suspected), hepatitis C antibodies, and HIV antibodies, with HIV screening conducted per the Uganda Ministry of Health HIV testing algorithm [28].

Urine dipstick tests screened for glucose, blood, white blood cells, nitrite, and protein. Volunteers with grade 1 abnormalities in haematology, biochemistry, or urinalysis (per FDA toxicity grading scale for preventive vaccine clinical trials [29]) were retested once. Those with normal repeat results could participate at the investigator’s discretion, while those with persistent abnormalities were excluded and referred for management if needed. Female participants underwent a urine pregnancy test for human chorionic gonadotrophin (HCG).

### 2.3. SARS-CoV-2 Serology Screening

**Figure 1:**
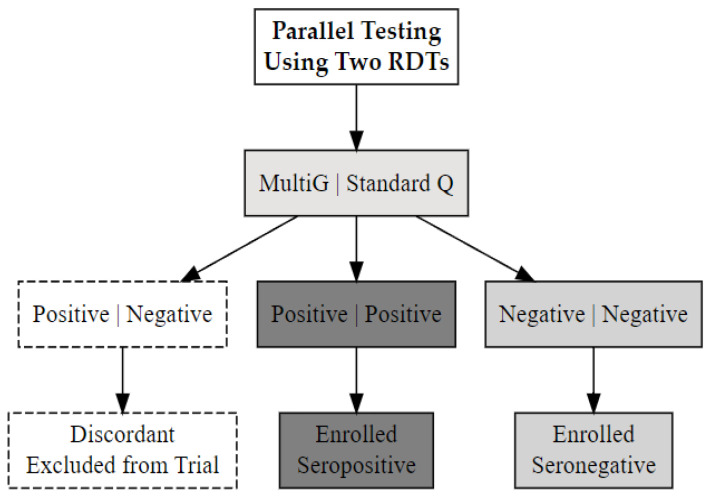
The SARS-CoV-2 serostatus determination process using two validated rapid serology test kits, Multi G (MGFT3) and Standard Q (COVID-19 IgM/IgG Plus).

Blood samples obtained by venepuncture were tested using two SARS-CoV-2 serology rapid test kits: (i) Multi G (MGFT3), Multi-G bvba, Antwerpen, Belgium; (ii) Standard Q (Standard Q COVID-19 IgM/IgG Plus), SD Biosensor, Inc., Suwon-si, Republic of Korea. Both kits, which detect IgM and IgG antibodies to SARS-CoV-2 in serum, plasma, or whole blood, demonstrated ≥98% specificity and sensitivity in a validation study in Uganda [30]. Participants were classified as SARS-CoV-2 seropositive if both test kits detected antibodies and seronegative status if neither did. Those with discordant results (positive on one kit, negative on the other) were categorised as having indeterminate serostatus and excluded from the trial (Figure 1). The stored enrolment samples were retested using rapid tests and ELISA, with the participants’ final SARS-CoV-2 serostatus determined from these results.

### 2.4. Eligibility Assessment and Procedures at Enrolment

At the enrolment visit, a study clinician confirmed eligibility by reviewing screening results, updating medical history, assessing COVID-19 vaccination status, medications, and contraceptive use, and conducting a repeat physical examination. Female participants underwent a pregnancy test, with only those testing negative proceeding to enrolment. The eligible participants were then enrolled, had blood samples collected for safety and immunogenicity assessments, and received the first vaccine dose.

### 2.5. Procedures for Assessing Safety

Local and systemic solicited adverse events were monitored after each vaccination. Participants remained at the clinic for up to 60 min post-vaccination to observe any immediate reactions. They were given a vaccine diary card to record and grade adverse events occurring within seven days. A study nurse reviewed the vaccine diary card with the participants, providing instructions on how to complete it. Blood samples were collected at each visit for safety evaluations, and appropriate action was taken for abnormal results. Vital signs were measured at each visit, along with physical examinations, including injection site assessments, on the day of vaccination and one week later. Symptom-directed physical examinations were conducted at follow-up visits. The participants were routinely asked about COVID-19 symptoms and instructed to report any symptoms to facilitate timely SARS-CoV-2 testing. Unsolicited adverse events were documented at each study visit and via telephone follow-up two days after vaccination, with study doctors recording diagnoses, symptoms, onset and resolution dates.

### 2.6. Procedures for Assessing Primary Immunogenicity Endpoint

Blood samples were collected at weeks 0, 1, 2, 4, 6, 8, 12, and 24 to assess immune responses to the vaccine (File S2). The primary outcomes included serum IgG antibodies to the SARS-CoV-2 S glycoprotein, measured by ELISA two weeks after the first and second vaccinations, although serum IgG antibody levels were tested at all timepoints. Binding IgG antibodies to the SARS-CoV-2 spike glycoprotein were quantified using a validated in-house ELISA, as previously described [31]. Briefly, medium-binding 96-well plates (Greiner Bio-One; Kremsmünster, Austria; #655001) were coated overnight at 4 °C with 3.0 µg/mL of recombinant wild-type spike antigen (R&D Systems; Minneapolis, MN, USA; #10474-CV-01M) in PBS. The plates were washed with PBS-T (0.05% Tween-20) and blocked with 1% BSA in PBS-T for 1 h at room temperature. Heat-inactivated plasma samples (56 °C, 30 min) were diluted 1:100 in blocking buffer and added in duplicate for 2 h. After washing, the plates were incubated with horseradish peroxidase–conjugated goat anti-human IgG (γ-chain specific, Sigma-Aldrich; St. Louis, MO, USA; #A0170; 1:10,000) for 1 h. Detection was performed using TMB substrate (SeraCare; Milford, MA, USA; #5120-0075), stopped after 3 min with 1 M HCl (SeraCare, #5150-0021), and optical density was read at 450 nm. Background signals were corrected using blank wells. Antibody concentrations were derived from a 4-parameter logistic standard curve and expressed in ng/mL. The lower limit of quantification (LLOQ) of this assay was defined as OD 0.432, which equated to 1000 ng/mL; values below lower limit of quantification (LLOQ) threshold were assigned 0 ng/mL.

Functional antibody responses were assessed by a pseudovirus neutralisation assay (PNA) two weeks after the second vaccination. The pseudovirus neutralisation assays were conducted as previously described in detail in Pollock et al. 2022 [25]. Further details can also be found in the Supplementary Information (File S3). All assays were performed at the MRC/UVRI and LSHTM Uganda Research Unit laboratories in Entebbe, Uganda.

### 2.7. Statistical Methods

The sample size calculation aimed to detect a difference of 0.7 on the log10 IC50 scale (corresponding to a slope of 1.4) for SARS-CoV-2 neutralisation at six weeks (two weeks post-second vaccination) between seropositive and seronegative participants, with a 97% power (2α = 0.05) and an estimated standard deviation of approximately 1.5 for neutralisation log10 IC50 values. Data were captured in electronic case report forms using REDCap software (Westlake, TX, USA, version 14.5.8) and transferred to Stata 18.0 (StataCorp, College Station, TX, USA) for cleaning and analysis. A CONSORT flow diagram was used to illustrate participant enrolment, follow-up, and analysis. Baseline characteristics and safety outcomes were summarised as counts and percentages and compared between arms using Fisher’s exact test. Given the skewed distribution of the neutralisation data, an offset from zero was added to the markers before the analysis. Linear mixed-effects models, with a random participant term and adjustments for age and sex, were used for data analysis.

## 3. Results

**Figure 2:**
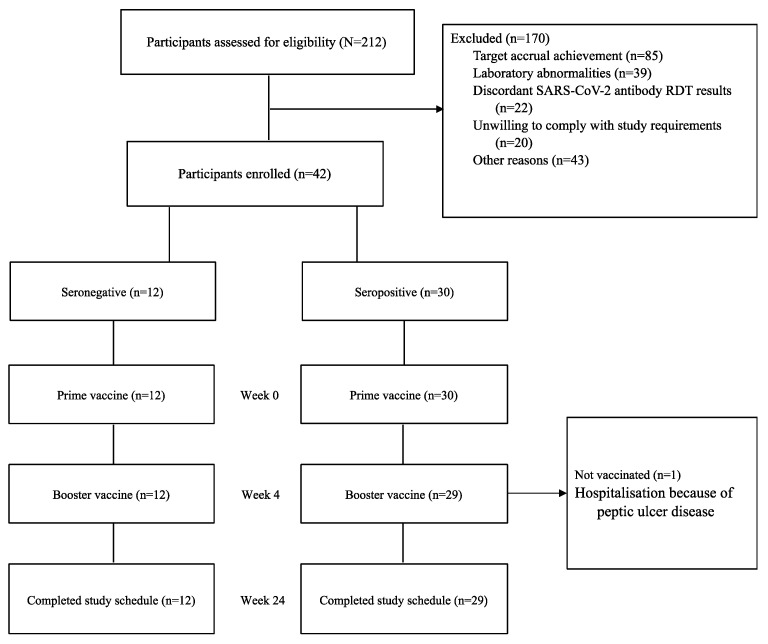
COVAC Uganda trial profile.

A total of 212 participants (51% male, n = 109) were screened between December 2021 and April 2022. Of these, 42 participants were enrolled, with 21 initially classified as seronegative and 21 as seropositive for SARS-CoV-2. Exclusion reasons included closure of enrolment after achieving target accrual (n = 85), laboratory abnormalities (n = 39), discordant SARS-CoV-2 antibody rapid test results (n = 22), unwillingness to comply with study requirements (n = 20), and other reasons (n = 43), as shown in Figure 2 (trial profile). Repeat screening of enrolment samples revealed seroconversion in nine initially seronegative participants, resulting in 30 being assigned to the seropositive arm and 12 to the seronegative arm.

The demographic characteristics of the enrolled participants (Table 1). The mean age was 30.2 (SD ± 8.3) years. The distribution of participants characteristics was similar across both arms.

### 3.1. Reactogenicity

Systemic reactions were similar across study arms following both the prime and booster vaccinations. The most common reactions following the prime vaccination were fatigue/malaise (47.6%), headache (42.9%) and chills/shivering (40.1%). After the booster, these reactions occurred more frequently: fatigue/malaise (63.4%), headache (61.0%), and chills/shivering (58.5%). No grade 3 or higher systemic reactions were reported following the prime vaccination, but one participant in the seropositive arm experienced ≥grade 3 chills/shivering and headache after the booster. Local reactions, mostly grade 1 and 2, were comparable between arms, with pain (71.4%) and tenderness (66.7%) being the most common after the prime vaccination. No erythema or swelling was reported. Comparable reactions and frequencies were observed after the booster vaccination. A summary of reactogenicity events is provided in Table 2.

### 3.2. Other Adverse Events

One serious adverse event, a prolonged hospitalization due to peptic ulcer disease exacerbation in a SARS-CoV-2 seropositive participant, was reported. While the event was considered unlikely to be related to vaccination, the Trial Steering Committee advised against a booster dose, citing the participant’s ineligibility due to active disease and the inability to fully exclude vaccine-related exacerbation. Unsolicited clinical adverse events were more common after the booster dose (n = 137) than after the prime dose (n = 32), with similar distribution across seropositive and seronegative arms.

Grade 3 or higher laboratory abnormalities were more frequent after the second vaccination than the first (39 vs. 9) (Table 3), with neutropenia, lymphopenia, and glucose abnormalities being most common. These abnormalities were more prevalent in the SARS-CoV-2 seropositive arm compared to the SARS-CoV-2 seronegative arm after both the first (7 vs. 2) and the second vaccinations (27 vs. 12), with notable differences in thrombocytopenia (first: 4 vs. 0; second: 8 vs. 0). None of the grade 3 or higher clinical AEs or laboratory abnormalities were attributed to the vaccine.

## 4. Immunogenicity

### 4.1. Significant Elevation of Spike-Specific IgG Binding Antibodies Following Two Vaccinations

**Figure 3:**
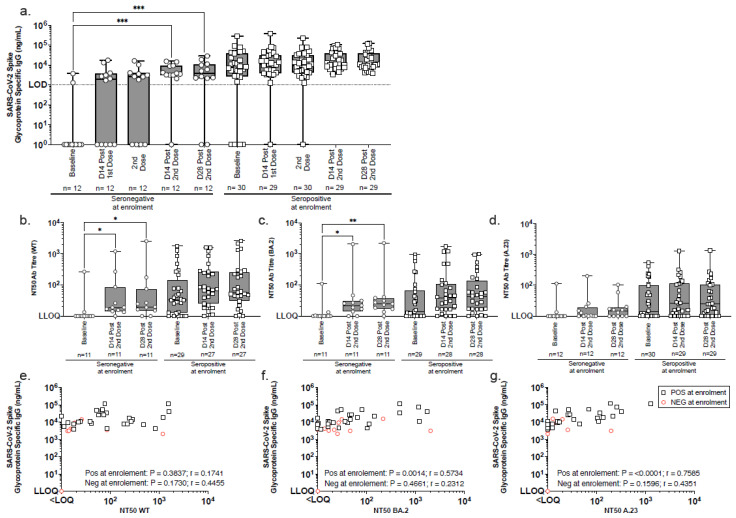
Longitudinal analysis of SARS-CoV-2 spike-specific IgG and neutralising antibody responses by SARS-CoV-2 serostatus. (**a**) SARS-CoV-2 spike-binding IgG were measured by ELISA in serum samples collected at baseline, 14 days post-1st immunisation, at 2nd immunisation, and at 14 and 28 days post-2nd immunisation. Participants were stratified enrolment sero-status: seronegative (circles) and seropositive (squares). (**b**–**d**) Pseudoneutralisation assays assessed neutralisation activity against (**b**) wild-type (WT), (**c**) BA.2, and (**d**) A.23.1 pseudoviruses using serum samples from baseline and days 14 and 28 post-2nd immunisation. The box and whisker plots illustrate the median values, interquartile range (IQR), and minimum/maximum values. (**e**–**g**) Comparative analyses of SARS-CoV-2 Spike IgG binding ELISA and pseudo neutralisation assay data for (**e**) WT, (**f**) BA.2, and (**g**) A.23.1 viruses were conducted using serum samples collected 14 days post-second immunisation. Group comparisons were conducted using the Friedman test with Dunn’s correction for multiple comparisons (**a**–**d**) and Spearman rank correlation for associations (**e**–**g**). Significance levels: * *p* < 0.05; ** *p* <0.01; *** *p* <0.001. LLOQ = lower limit of quantification. LOD = limit of detection.

SARS-CoV-2 spike-specific IgG antibodies increased significantly after two vaccinations, as evidenced by the serum IgG binding antibody concentrations measured by ELISA at baseline and two-weeks post-immunisation in 42 participants (Figure 3a). Among 12 seronegative participants at enrolment, 91.6% (11/12) developed IgG responses. The median IgG concentration rose from 0 ng/mL at baseline to 3695 ng/mL (IQR 3101–9109) at 14 days post-second dose (*p* = 0.0003 at 14 days; *p* = 0.0001 at 28 days) (Figure 3a, Table 4). Of the two seronegative individuals shown in Figure 3a with spike-specific IgG levels below the limit of quantification on day 28 following the second dose, one individual remained seronegative across all timepoints tested and did not mount a detectable immune response to vaccination. The second individual exhibited a transient IgG response, peaking at 2075 ng/mL on day 14 post-second dose, which would not be considered a strong IgG response. Therefore, the decline to undetectable levels by day 28 post-second dose is not considered unexpected. All initially seropositive participants remained so post-vaccination, with median IgG levels rising from 7496 ng/mL (IQR 2662–38,969) at baseline to 11,028 ng/mL (IQR 7828–37,563) at 14 days post-second dose (Figure 3a, Table 4). Although this approximately two-fold increase was not statistically significant, it suggests a boosting effect. These findings highlight the vaccine’s strong immunogenicity in seronegative individuals and its potential to enhance pre-existing immunity (Figure 3, Table 4).

### 4.2. Improved Neutralising Antibody Response Post-Second Vaccination Across Multiple SARS-CoV-2 Variants

Neutralising activity of serum antibodies was assessed using a pseudoneutralisation assay with circulating SARS-CoV-2 variants [wild-type (WT), BA.2, and A.23.1]. The assays were conducted on serum samples collected at baseline, 14 and 28 days after the second vaccination. Among the seronegative participants at enrolment, the median NT50 neutralising titres 14 days after vaccination were as follows: WT (19; IQR 14–85), BA.2 (22; IQR 14–31), and A.23.1 [<limit of quantification (LOQ); IQR < LOQ-19]. Significant increases in neutralisation titres were observed for WT (*p* = 0.0120 and *p* = 0.0315) and BA.2 (*p* = 0.0315 and *p* = 0.0013) at 14 and 28 days (Figure 3b–d, Table 5). Although A.23.1 neutralisation remained low, notable response rates were observed from 2/11 to 10/11 for WT, from 3/11 to 9/11 for BA.2, and from 2/12 to 7/12 for A.23.1, indicating an overall improvement post-vaccination.

Among seropositive participants, neutralising antibody titres (NT50) increased against all variants 14 days after the second vaccine dose, though these changes were not statistically significant. The median NT50 values rose from 32 (IQR 12–143) to 73 (IQR 25–264) for WT, from 14 (IQR < LOQ-66) to 39 (IQR 16–110) for BA.2, and from 14 (IQR < LOQ-101) to 26 (IQR < LOQ-114) for A.23.1 (Figure 3b–d, Table 5). The proportion of participants with detectable neutralising responses also increased: for WT, from 79.3% (23/29) at baseline to 96.3% (26/27) post-immunisation, for BA.2, from 62.1% (18/29) to 89.3% (25/28); and for A.23.1, from 63.3% (19/30) to 79.3% (23/29). A significant correlation between SARS-CoV-2 serum IgG levels and neutralising activity was found in seropositive participants post-second dose, particularly for BA.2 (*p* = 0.0014) and A.23.1 (*p* < 0.0001) (Figure 3e–g).

The results suggest enhanced neutralising antibody responses post-vaccination, particularly against the WT and BA.2 variants, with broader activity, including A.23.1. The data demonstrate the vaccine’s ability to boost neutralising antibody levels in both seronegative and seropositive individuals, emphasising its potential to enhance immune protection across diverse SARS-CoV-2 variants.

Geometric mean (GM) and adjusted geometric mean (aGM) titres of spike-specific IgG-binding and neutralising antibody responses, stratified by SARS-CoV-2 serostatus (Table 5). Data cover two weeks post–first and second vaccine doses. aGM values, adjusted for baseline antibody levels, sex, and age, compare responses between seropositive and seronegative participants. ‘ND’ denotes unavailable data. The aGM titres illustrate the impact of prior SARS-CoV-2 exposure on vaccine-induced immunity.

The aGM of spike-specific IgG binding antibodies was significantly higher in the seropositive group compared to the seronegative group two weeks post-vaccination, after both the first (aGM: 1.72, 95% CI: 1.06–2.37) and the second dose (aGM: 1.41, 95% CI: 0.87–1.94). Similarly, the aGM for nucleocapsid-specific IgG was higher in seropositive participants, reflecting prior exposure. However, neutralising antibody titres did not differ significantly between groups across the three variants tested after the second dose (Table 5).

## 5. Discussion

We present findings from COVAC Uganda, a phase 1 trial evaluating the safety and immunogenicity of LNP-nCOV saRNA-02, a saRNA vaccine encoding the SARS-CoV-2 S glycoprotein, in seronegative and seropositive Ugandan participants. To our knowledge, this is the first saRNA vaccine trial reported from Africa.

Our findings demonstrate that the vaccine was safe and well tolerated, with mostly mild to moderate transient reactogenicity. Similarly, the UK-based COVAC1 phase 1 trial, which evaluated a similar saRNA vaccine, demonstrated its safety and tolerability. COVAC1, a dose-finding trial, administered doses from 0.1 μg to 10.0 μg, with a booster at the same dose after four weeks. However, moderately severe reactogenicity events were more frequent in COVAC1 than in COVAC Uganda.

A phase 2a trial also in the UK, which included a more diverse demographic, with older participants and individuals with co-morbidities, evaluated the same vaccine at fixed doses of 1 μg (prime) and 10 μg (boost) administered 14 weeks apart. That study did not find any safety concerns [26]. However, tolerability was dose-dependent, with higher frequency and severity of adverse reactions after the 10 μg dose, where 17% of recipients experienced grade 3 adverse events. In both UK trials, adverse reactions were more common in the younger participants, a trend not observed in COVAC Uganda, likely due to a less diverse age profile.

In our trial, reactogenicity was similar among participants with and without prior infection, with only mild to moderate local and systemic reactions reported. Thrombocytopenia occurred more frequently after the boost dose, particularly in the seronegative arm, but all cases were asymptomatic and resolved before follow-up completion. Thrombocytopenia has been observed with other COVID-19 vaccines, in which some cases presented with symptoms [32,33]. This study recorded one serious adverse event: hospitalization for exacerbated peptic ulcer disease in the SARS-CoV-2 seropositive arm after the prime dose, considered unlikely to be vaccine-related.

The vaccine elicited strong humoral responses in SARS-CoV-2 seronegative participants, with 91.6% developing spike-specific IgG antibodies 14 days after the boost. Antibody levels in these individuals matched or exceeded those in seropositive individuals, highlighting the vaccine’s ability to prime naïve immune systems. These findings align with evidence that saRNA has potential to elicit robust humoral responses in unexposed populations [34]. However, durability remains uncertain, as data from other platforms suggest neutralising antibodies may decline within six months following vaccination [35,36]. Given the rapid evolution of SARS-CoV-2 variants, antibody longevity and breadth are key considerations for future vaccine design [37,38].

SARS-CoV-2 seropositive participants exhibited a moderate antibody boost, reinforcing pre-existing immunity, consistent with findings from other COVID-19 vaccines [39]. Despite higher baseline antibody levels, their post-vaccination increase was less pronounced than in seronegative participants, likely due to a ceiling effect [39]. The nearly two-fold increase in IgG levels, though not statistically significant, suggests a strong boost response.

During the development of the protocol, it was decided that we would use the MultiG and StandardQ rapid tests to screen participants at enrolment for SARS-CoV-2 IgG and IgM antibodies. The results from these tests were used to group the participants according to serostatus. We acknowledge the discrepancy for two individuals in the results obtained using two “rapid-test” assays at enrolment (MultiG/StandardQ) and the in-house ELISA for assessment of IgG-specific SARS-CoV-2 spike protein used to monitor immune responses during the trial. The baseline samples from these two individuals were classified as “sero-negative” using Rapid Diagnostics Tests (RDTs), as specified in the protocol, with one value very close to the 1000 ng/mL assay cut-off (~1200 ng/mL) and the other ~4000 ng/mL (considered low) in the ELISA. This discrepancy probably reflects inherent and documented differences the sensitivities/specificities of these very different assays; the rapid tests measure IgM and IgG, whereas the ELISA measures IgG only, for example, with the latter also including an amplification step. Whilst we acknowledge this as a study limitation, both samples in question did not demonstrate neutralization in any of the pseudoneutralisation assays. Furthermore, both samples tested negative in our validated SARS-CoV-2 nucleocapsid IgG ELISA.

The lack of a significant difference in neutralising antibody titres between seropositive and seronegative groups, despite higher binding antibody levels, suggests a potential dissociation between humoral response and neutralisation capability. This stems from the spike glycoprotein’s antigenic structure, which induces binding but not necessarily neutralising antibodies [40]. While the saRNA vaccine elicits strong humoral responses, further research is needed to fully elucidate its functional protective mechanisms.

Interestingly, the markedly lower neutralization titers against A.23.1 compared to BA.2 in this vaccinated cohort contrast with our previous findings in a more urban, mobile population, where A.23.1 priming was dominant. Several factors may account for this discrepancy. First, the trial was conducted after A.23.1 had ceased circulating, limiting natural boosting prior to enrolment. Second, the cohort was drawn from a rural population with potentially lower exposure intensity during the A.23.1 wave. Notably, the vaccine used was based on the ancestral strain, which may explain the relatively higher responses to the prototype virus. Third, recruitment coincided with the Omicron wave, likely leading to enhanced priming against Omicron variants in a subset of participants. This is supported by our screening data showing seroconversion in several individuals prior to vaccination, suggesting recent exposure. Together, these factors may explain the comparatively muted A.23.1 responses and underscore the importance of considering timing, exposure dynamics, and baseline immunity when interpreting variant-specific vaccine responses [14].

The strong immune responses observed in our study contrast sharply with the results observed in the UK trials, where similar saRNA vaccines elicited weaker responses [26]. This difference may partly be attributable to the inclusion of the ORF4a gene, which could modulate immune responses [41]. Ongoing investigations, including a transcriptomics study, aim to further characterise the innate immunity and T-cell responses in this Ugandan cohort.

saRNA technology is still novel, with few vaccines assessed globally. The first approved saRNA vaccine, ARCT-154 (CSL and Arcturus Therapeutics), received approval in Japan in November 2023 based on a phase 3 trial demonstrating superior immunogenicity and safety over BNT162b2 (Pfizer-BioNTech) mRNA COVID-19 vaccine [42]. Our findings support the immunogenic potential of saRNA platforms to elicit high antibody titres with small doses due to their self-amplifying nature.

A potential limitation of this study is the small sample size, which may affect the generalizability of the results. However, the consistent trends observed in both seronegative and seropositive participants provide valuable insights into the immunogenic potential of saRNA vaccines, particularly in an African population where vaccine trials remain limited. Larger and more diverse studies are needed to validate these findings. Secondly, the 42-day screening period led to some participants who tested SARS-CoV-2 negative acquiring the virus before enrolment, as confirmed by repeat testing. This resulted in an imbalance between the two groups, with a higher number of seropositive than seronegative individuals. Additionally, although IgM is known to contribute to early neutralizing responses, its evaluation was not undertaken due to resource constraints at the time of analysis, a limitation we acknowledge, as it may have restricted a more comprehensive understanding of the early-phase humoral response. Lastly, the absence of a placebo group limits the ability to attribute all observed effects solely to the vaccine.

## 6. Conclusions

In conclusion, this study provides important evidence of the immunogenicity of a novel saRNA-based COVID-19 vaccine in an African population, showing strong induction of spike-specific binding antibodies in both seronegative and seropositive individuals. While binding antibody responses were robust, the relatively modest neutralising antibody responses suggest the potential for further optimisation of the vaccine platform. These findings enhance the understanding of saRNA vaccines and highlight their potential role in priming naïve immune systems and boosting pre-existing immunity, offering important insights for future vaccine development and pandemic preparedness.

## Figures and Tables

**Table 1:** Demographic characteristics of participants enrolled in the COVAC Uganda trial.

Characteristics	SARS-CoV-2 Seropositive (n = 30) n (%)	SARS-CoV-2 Seronegative (n = 12) n (%)	*p*-Value
**Age (years), mean (SD)**	30.9 (8.0)	28.4 (9.1)	
Age group			0.753
18–24	10 (33.3)	5 (41.7)	
25–34	8 (26.7)	4 (33.3)	
35–45	12 (40.0)	3 (25.0)	
Gender			0.180
Male	15 (50.0)	9 (75.0)	
Female	15 (50.0)	3 (25.0)	
Contraception use			0.311
Yes	19 (63.3)	6 (50.0)	
No	11 (36.7)	6 (50.0)	
Type of contraceptive			0.766
Injectable	6 (31.6)	2 (33.3)	
Implant	9 (47.4)	2 (33.3)	
Intra uterine device	1 (5.3)	0 (0.0)	
Oral	1 (5.3)	0 (0.0)	
Other	2 (10.5)	2 (33.3)	
Ever smoked			1.000
Never	28 (93.3)	12 (100.0)	
Yes, currently	0 (0.0)	0 (0.0)	
Yes, previously	2 (6.7)	0 (0.0)	

**Table 2:** Local and systemic reactogenicity events following prime and booster vaccination by SARS-CoV-2 serostatus.

	Post-Prime Vaccination									Post-Boost Vaccination								
Event	SARS-CoV-2 Seropositive (n = 30)				SARS-CoV-2 Seronegative (n = 12)				Total (N = 42)	SARS-CoV-2 Seropositive (n = 29)				SARS-CoV-2 Seronegative (n = 12)				Total (N = 41)
Grade	One n (%)	Two n (%)	Three+ n (%)	All n (%)	One n (%)	Two n (%)	Three+ n (%)	All n (%)	N (%)	One n (%)	Two n (%)	Three+ n (%)	All n (%)	One n (%)	Two n (%)	Three+ n (%)	All n (%)	N (%)
Systemic																		
Chills /Shivering	9 (30.0)	2 (6.7)	0 (0.0)	11 (36.7)	5 (41.7)	1 (8.3)	0 (0.0)	6 (50.0)	17 (40.5)	10 (34.5)	6 (20.6)	1 (3.4)	17 (58.6)	7 (58.3)	0 (0.0)	0 (0.0)	7 (58.3)	24 (58.5)
Myalgia	5 (16.7)	2 (6.7)	0 (0.0)	7 (23.3)	4 (33.3)	0 (0.0)	0 (0.0)	4 (33.3)	11 (26.2)	10 (34.5)	2 (6.8)	0 (0.0)	14 (48.3)	3 (25.0)	0 (0.0)	0 (0.0)	3 (250)	15 (36.5)
Arthralgia	5 (16.7)	2 (6.7)	0 (0.0)	7 (23.3)	6 (50.0)	0 (0.0)	0 (0.0)	6 (50.0)	13 (30.9)	8 (27.5)	2 (6.8)	0 (0.0)	10 (34.5)	4 (33.3)	0 (0.0)	0 (0.0)	3 (25.0)	14 (34.1)
Fatigue	11 (36.7)	2 (6.7)	0 (0.0)	13 (43.3)	7 (58.3)	0 (0.0)	0 (0.0)	7 (58.3)	20 (47.6)	12 (41.3)	5 (17.2)	0 (0.0)	17 (58.6)	8 (66.7)	1 (8.3)	0 (0.0)	9 (75.0)	26 (63.4)
Headache	7 (23.3)	2 (6.7)	0 (0.0)	9 (30.0)	7 (58.3)	2 (16.7)	0 (0.0)	9 (75.0)	18 (42.9)	12 (41.3)	6 (206)	1 (3.4)	19 (65.5)	4 (33.3)	2 (16.7)	0 (0.0)	6 (50.0)	25 (60.9)
Nausea	4 (13.3)	0 (0.0)	0 (0.0)	4 (13.3)	4 (33.3)	0 (0.0)	0 (0.0)	4 (33.3)	8 (19.0)	6 (20.6)	2 (6.8)	0 (0.0)	8 (27.6)	4 (333)	0 (0.0)	0 (0.0)	4 (33.3)	12 (29.2)
Vomiting	0 (0.0)	0 (0.0)	0 (0.0)	0 (0.0)	1 (8.3)	0 (0.0)	0 (0.0)	0 (8.3)	0 (0.0)	0 (0.0)	2 (6.8)	0 (0.0)	2 (6.8)	0 (0.0)	0 (0.0)	0 (0.0)	0 (0.0)	2 (4.8)
Any	25 (83.3)	5 (16.7)	0 (0.0)		10 (83.3)	3 (25.0)	0 (0.0)		27 (64.3)	20 (68.9)	12 (41.4)	1 (3.4)		10 (83.3)	3 (25.0)	0 (0.0)		35 (85.4)
Local																		
Pain	17 (56.7)	3 (10.0)	0 (0.0)	20 (66.7)	8 (66.7)	2 (16.7)	0 (0.0)	10 (83.3)	30 (71.4)	14 (48.2)	6 (20.6)	1 (3.4)	21 (72.4)	6 (50.0)	1 (8.3)	0 (0.0)	7 (58.3)	28 (68.2)
Tenderness	14 (46.7)	6 (20.0)	0 (0.0)	20 (66.7)	8 (66.7)	0 (0.0)	0 (0.0)	8 (66.7)	28 (66.7)	12 (41.3)	7 (24.1)	0 (0.0)	19 (65.5)	6 (50.0)	1 (8.3)	0 (0.0)	7 (58.3)	26 (63.4)
Erythema	0 (0.0)	0 (0.0)	0 (0.0)	0 (0.0)	0 (0.0)	0 (0.0)	0 (0.0)	0 (0.0)	0 (0.0)	0 (0.0)	0 (0.0)	0 (0.0)	0 (0.0)	0 (0.0)	0 (0.0)	0 (0.0)	0 (0.0)	0 (0.0)
Swelling	0 (0.0)	0 (0.0)	0 (0.0)	0 (0.0)	0 (0.0)	0 (0.0)	0 (0.0)	0 (0.0)	0 (0.0)	0 (0.0)	0 (0.0)	0 (0.0)	0 (0.0)	0 (0.0)	0 (0.0	0 (0.0)	0 (0.0)	0 (0.0)
Any	20 (66.6)	6 (20.0)	0 (0.0)		10 (83.3)	2 (16.7)	0 (0.0)		35 (83.3)	17 (58.6)	8 (27.6)	1 (3.4)		6 (50.0)	1 (8.3)	0 (0.0)		31 (75.6)

**Table 3:** Frequency of ≥grade 3 laboratory adverse events following prime and booster vaccination by SARS-CoV-2 status.

Event	Post-Prime Vaccination			Post-Boost Vaccination		
	SARS-CoV-2 Seropositive	SARS-CoV-2 Seronegative	All	SARS-CoV-2 Seropositive	SARS-CoV-2 Seronegative	All
	≥Grade 3	≥Grade 3	≥Grade 3	≥Grade 3	≥Grade 3	≥Grade 3
Raised creatinine	0	0	0	0	0	0
Raised ALT	0	0	0	0	0	0
Raised AST	0	0	0	0	0	0
Raised ALP	0	0	0	0	0	0
Raised bilirubin	0	0	0	0	0	0
Raised GGT	0	0	0	0	0	0
Hypoglycemia	1	0	1	1	0	1
Hyperglycemia	0	1	1	0	1	1
Anaemia	0	0	0	0	0	0
Leukopenia	0	0	0	0	0	0
Leukocytosis	0	0	0	0	0	0
Neutropenia	2	1	3	8	5	13
Lymphopenia	0	0	0	10	6	16
Thrombocytopenia	4	0	4	8	0	8
All	7	2	9	27	12	39

ALT, alanine aminotransferase; AST, aspartate transferase; ALP, alkaline phosphatase; GGT, gamma-glutamyl transferase.

**Table 4:** Binding and functional neutralising antibody responses stratified by serostatus at enrolment (<LOQ = below limit of quantification).

		*Baseline*	*D14 Post 1st Dose*	*2nd Dose*	*D14 Post 2nd Dose*	*D28 Post 2nd Dose*	*Baseline*	*D14 Post 1st Dose*	*2nd Dose*	*D14 Post 2nd Dose*	*D28 Post 2nd Dose*
SARS-CoV-2 Spike IgG ELISA (ng/mL)	*No. participants*	12	12	12	12	12	29	28	29	29	29
	*Minimum*	<LOQ	<LOQ	<LOQ	<LOQ	<LOQ	<LOQ	<LOQ	<LOQ	3873	3411
	*25% Percentile*	<LOQ	<LOQ	<LOQ	3101	2188	2662	3948	4257	7828	7277
	*Median*	<LOQ	1869	2601	3695	3831	7496	11,198	9204	11,028	11,010
	*75% Percentile*	<LOQ	3736	3946	9109	10,781	38,969	30,382	31,943	37,563	40,163
	*Maximum*	3686	17,353	16,115	15,373	28,303	282,434	377,800	219,842	118,877	102,458
WT pseudoneutralisation (NT50)	*No. participants*	11			11	11	29			27	27
	*Minimum*	<LOQ			<LOQ	<LOQ	10			<LOQ	<LOQ
	*25% Percentile*	<LOQ			14	15	12			25	30
	*Median*	<LOQ			19	20	32			73	57
	*75% Percentile*	<LOQ			85	74	143			264	261
	*Maximum*	265			1193	2537	1782			1578	2612
BA.2 pseudoneutralisation (NT50)	*No. participants*	11			11	11	29			28	28
	*Minimum*	<LOQ			<LOQ	<LOQ	<LOQ			<LOQ	<LOQ
	*25% Percentile*	<LOQ			14	17	<LOQ			16	17
	*Median*	<LOQ			22	25	14			39	43
	*75% Percentile*	10			31	38	66			110	139
	*Maximum*	109			2038	2223	956			1726	986
A.23.1 pseudoneutralisation (NT50)	*No. participants*	12			12	12	30			29	29
	*Minimum*	<LOQ			<LOQ	<LOQ	<LOQ			<LOQ	<LOQ
	*25% Percentile*	<LOQ			<LOQ	<LOQ	<LOQ			<LOQ	<LOQ
	*Median*	<LOQ			<LOQ	14	14			26	24
	*75% Percentile*	<LOQ			19	19	101			114	104
	*Maximum*	113			199	105	536			1293	1359

**Table 5:** Comparison of post-vaccination geometric mean concentrations of spike-specific IgG (ng/mL) and neutralising (NT50) antibodies by SARS-CoV-2 serostatus.

Marker	Two Weeks Post Dose 1				Two Weeks Post Dose 2			
	SARS-CoV-2 Positive	SARS-CoV-2 Negative			SARS-CoV-2 Positive	SARS-CoV-2 Negative		
	GM (95% CI)	GM (95% CI)	aGM (95% CI)	*p*-Value	GM (95% CI)	GM (95% CI)	aGM (95% CI)	*p*-Value
Spike-specific IgG by ELISA	4.00 (3.71, 4.29)	2.55 (1.69, 3.42)	1.72 (1.06, 2.37)	<0.001	4.20 (4.05, 4.37)	3.49 (2.97, 4.01)	1.41 (0.87, 1.94)	<0.001
Neutralising antibody (WT_NT_50_)	ND	ND	-	-	4.25 (3.61, 4.89)	3.87 (2.87, 4.87)	0.44 (−0.54, 1.42)	0.382
Neutralising antibody (A.23.1_NT_50_)	ND	ND	-	-	3.07 (2.11, 4.03)	3.79 (3.22, 4.35)	0.72 (−0.22, 1.65)	0.133
Neutralising antibody (BA.2_NT_50_)	ND	ND	-	-	3.89 (3.28, 4.49)	3.86 (2.77, 4.95)	0.35 (−0.68, 1.38)	0.502
Nucleocapsid-specific IgG	ND	ND	-	-	3.55 (3.21, 3.89)	1.65 (0.95, 2.34)	2.12 (1.57, 2.67)	<0.001

GM—Geometric mean, aGM—adjusted geometric mean comparing post-vaccination antibody concentration levels between SARS-CoV-2 positive and negative participants, adjusted for baseline value, sex, and age. ND: no data.

## Data Availability

The raw data supporting the conclusions of this article will be made available by the authors upon request from the corresponding author (JK).

## Supplementary Materials

The following supporting information can be downloaded at: https://www.mdpi.com/article/10.3390/vaccines13060553/s1, File S1: Structure of our second generation self amplifying RNA vaccine where the ORF4a gene has been inserted; File S2: schedule of study procedures; File S3: Conduct of ELISA and pseudovirus neutralisation assays (PNA) ELISA.
