# Supplementary material for: Safety and Immunogenicity of a Modified Self-Amplifying Ribonucleic Acid (saRNA) Vaccine Encoding SARS-CoV-2 Spike Glycoprotein in SARS-CoV-2 Seronegative and Seropositive Ugandan Individuals
Source: Vaccines (Basel). 2025 May 23;13(6):553. doi: 10.3390/vaccines13060553 (PMC12197785; doi:10.3390/vaccines13060553)
Supplement: Supplementary file 1 [file vaccines-13-00553-s001.zip › File S1_Structure of our second generation self ampl.pdf]

Supplementary information S1: Structure of our second generation self amplifying RNA vaccine where the ORF4a gene has been inserted [1]

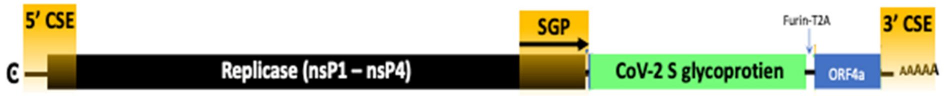

CSE, *Conserved Sequence Element*, nsP1, *Non-structural Protein 1*, nsP4, *Non structural Protein 4*, SGP Subgenomic promoter, CoV-2 Corona virus-2, 3'CSE, *3' Conserved Sequence Element*

Reference

1. Blakney AK, McKay PF, Bouton CR, Hu K, Samnuan K, Shattock RJ: **Innate Inhibiting Proteins Enhance Expression and Immunogenicity of Self-Amplifying RNA**. *Mol Ther* 2021, **29**(3):1174-1185.
