# Supplementary material for: Safety and Immunogenicity of a Modified Self-Amplifying Ribonucleic Acid (saRNA) Vaccine Encoding SARS-CoV-2 Spike Glycoprotein in SARS-CoV-2 Seronegative and Seropositive Ugandan Individuals
Source: Vaccines (Basel). 2025 May 23;13(6):553. doi: 10.3390/vaccines13060553 (PMC12197785; doi:10.3390/vaccines13060553)
Supplement: Supplementary file 1 [file vaccines-13-00553-s001.zip › File S2_Schedule of procedures.pdf]

Supplementary information S2: schedule of study procedures

S1: Trial Assessment Schedule for all individuals

**Trial Visit**	**V1**	**V2**	**V2a**	**V3**	**V4**	**V5**	**V5a**	**V6**	**V7**	**V8**	**V9**	**V10**
Visit Type (site or telephone)	Site	Site	Site	Site	Site	Site	Site	Site	Site	Site	Site	Site
Trial Week	–6	0	0	1	2	4	4	5	6	8	12	24
Trial Day	–	0	1	7	14	28	29	35	42	56	84	168
Windows (days)	–42 to –1	–	None	0 to +2	–2 to +2	–4 to +10	none	0 to +2	–2 to +2	–4 to +4	–7 to +7	–20 to +7
Informed consent	X											
Medical history demographics	X											
Eligibility assessment	X	X										
Physical examination	X	X		X		X		X				
Weight/height (BMI)	X											
Vital signs (BP, HR, O_2 saturation and oral temperature)	X	X	X	X	X	X	X	X	X	X	X	X
Concomitant medication	X	X	X	X	X	X	X	X	X	X	X	X
Group allocation		X										
Vaccination		X				X						
Issue diary card for AEs		X				X						
Review diary card for AEs			X	X			X	X				
Symptom-directed physical examination			X		X		X		X	X	X	X
Record COVID-19 symptoms, swab if COVID-19 suspected and take sample for DNA-PCR[1]	X	X	X	X	X	X	X	X	X	X	X	X
Record adverse events		X	X	X	X	X	X	X	X	X	X	X
HIV and HCV screen	~5 mL											
SARS-CoV-2 antibodies	~5 mL											

Laboratory safety tests[2]	~10 mL	~10 mL		~10 mL	~10 mL	~10 mL		~10 mL	~10 mL	~10 mL	~10 mL	~10 mL
Urine dipstick	X											
Urinary pregnancy test	X	X				X					X	
Blood for central serum immunogenicity assays		6 mL	6 mL	6 mL	6 mL	6 mL	6mL	6 mL	6 mL	6 mL	6 mL	6 mL
Blood for central cellular immunogenicity assays		60 mL	60 mL			60 mL	60 mL		60 mL		60 mL	
Blood for RNA paxgene tubes		2.5 ml	2.5 ml			2.5 ml	2.5 ml					
Blood volume (approx.)	20 mL	80.5 mL	68.5 mL	16 mL	16 mL	80.5 mL	68.5 mL	16 mL	76 mL	16 mL	76 mL	16 mL

1 If symptoms for COVID-19 present on V2 (enrolment visit), do not enrol until SARS-CoV-2 PCR results return as negative.

2 Haemoglobin, lymphocytes, neutrophils, platelets, creatinine, AST, ALT, ALP, total bilirubin, non-fasting glucose throughout. GGT at screening only.
