# Supplementary material for: Safety and Immunogenicity of a Modified Self-Amplifying Ribonucleic Acid (saRNA) Vaccine Encoding SARS-CoV-2 Spike Glycoprotein in SARS-CoV-2 Seronegative and Seropositive Ugandan Individuals
Source: Vaccines (Basel). 2025 May 23;13(6):553. doi: 10.3390/vaccines13060553 (PMC12197785; doi:10.3390/vaccines13060553)
Supplement: Supplementary file 1 [file vaccines-13-00553-s001.zip › File S3_ELISA and PVA Methods.pdf]

Supplementary information S3: Conduct of ELISA and a pseudovirus neutralisation assays (PNA)

ELISA

Sera were heat inactivated for 30 mins at 56°C prior to storage at -20°C before assessment in immunological assays. Binding antibody concentrations induced by the vaccine in participant sera were assessed using an anti-S IgG ELISA in 96-well plates coated with the stabilised SARS-CoV-2 spike protein in the pre-fusion conformation. Background optical density (OD) 450nm readings from uncoated wells (blank wells) were subtracted from readings in test wells. Vaccine-induced seroconversion to anti-S IgG was considered to have occurred in those where there was an OD of above 0.2nm in the ELISA. This threshold was set during optimisation of the ELISA using standards from National Institute for Biological Standards and Control (NIBSC), convalescent sera from individuals recovered from SARS-CoV-2 infection and sera taken prior to December 2019. Baseline convalescent sera were available from 32 individuals enrolled into the wider COVAC1 trial with either a history of mild or moderate COVID or previously unknown, asymptomatic SARS-CoV-2 infection. A positive control of pooled plasma samples from NIBSC was included in each assay plate. When it became available (December 2020), the first WHO international standard anti-SARS-CoV-2 immunoglobulin was added at a concentration of 2 BAU/mL as an additional control. It was determined that 2 BAU/mL was the equivalent of approximately 20,000 ng/mL.

Pseudovirus Neutralisation Assay

SARS-CoV-2 neutralisation assays were conducted using pseudotyped (PSV) viruses at the MRC/UVRI and LSHTM Uganda Research Unit laboratories in Entebbe, Uganda. Briefly for the PSV assay, pseudotyped SARS-CoV-2 lentiviruses were produced in HEK293T/17 cells using a SARS-CoV-2 spike plasmid, HIV-1 gag-pol plasmid and a firefly luciferase reporter. Participant sera were serially diluted and incubated with PSV viral supernatant for 1 hour at 37°C. HEK-ACE2 cells were then co-incubated with the sera and PSV for up to 96 hours at 37°C before measurement of the luciferase activity using the Steady-Glo Luciferase assay system (Promega, Madison, WI). IC50 neutralisation titres were calculated as the dilution at which relative luminescence was reduced by 50% compared with control. For the PSV assay, the First WHO International Standard for anti- SARS-CoV-2 immunoglobulin was included as a positive control, which was determined to have an IC50 neutralisation titre of approximately 1:3000.
